# An open-source tool for longitudinal whole-brain and white matter lesion segmentation

**DOI:** 10.1016/j.nicl.2023.103354

**Published:** 2023-03-02

**Authors:** Stefano Cerri, Douglas N. Greve, Andrew Hoopes, Henrik Lundell, Hartwig R. Siebner, Mark Mühlau, Koen Van Leemput

**Affiliations:** a Athinoula A. Martinos Center for Biomedical Imaging, Massachusetts General Hospital, Harvard Medical School, USA; b Danish Research Centre for Magnetic Resonance, Copenhagen University Hospital Amager and Hvidovre, Copenhagen, Denmark; c Department of Radiology, Harvard Medical School, USA; d Department of Neurology, Copenhagen University Hospital Bispebjerg and Frederiksberg, Copenhagen, Denmark; e Institute for Clinical Medicine, Faculty of Medical and Health Sciences, University of Copenhagen, Denmark; f Department of Neurology and TUM-Neuroimaging Center, School of Medicine, Technical University of Munich, Germany; g Department of Health Technology, Technical University of Denmark, Denmark

**Keywords:** Longitudinal segmentation, Whole-brain segmentation, Lesion segmentation, Generative models, FreeSurfer

## Abstract

In this paper we describe and validate a longitudinal method for whole-brain segmentation of longitudinal MRI scans. It builds upon an existing whole-brain segmentation method that can handle multi-contrast data and robustly analyze images with white matter lesions. This method is here extended with subject-specific latent variables that encourage temporal consistency between its segmentation results, enabling it to better track subtle morphological changes in dozens of neuroanatomical structures and white matter lesions. We validate the proposed method on multiple datasets of control subjects and patients suffering from Alzheimer’s disease and multiple sclerosis, and compare its results against those obtained with its original cross-sectional formulation and two benchmark longitudinal methods. The results indicate that the method attains a higher test–retest reliability, while being more sensitive to longitudinal disease effect differences between patient groups. An implementation is publicly available as part of the open-source neuroimaging package FreeSurfer.

## Highlights

• A method for whole-brain segmentation of longitudinal MRI scans.
• The method is adaptive to different scanners and MRI sequences.
• It does not put any constraints on the number or the timing of follow-up scans.
• It can also segment white matter lesions simultaneously.
• The method is publicly available as part of FreeSurfer.

## 1 Introduction

Longitudinal imaging studies, in which subjects are scanned repeatedly over time, have several advantages over cross-sectional studies. Accordingly, longitudinal neuroimaging studies have provided valuable insights into temporal changes in healthy brain development (Giedd et al., 1999, Evans et al., 2006, Group, 2012, Choe et al., 2013, Mills et al., 2021) and aging (Scahill et al., 2003, Tamnes et al., 2013), as well as from neurodegenerative diseases such as Alzheimer’s disease (AD) (Fox et al., 1996, Laakso et al., 1998, Du et al., 2001, Halliday, 2017) or multiple sclerosis (MS) (Audoin et al., 2006, Fisher et al., 2008). In most instances, a longitudinal study design increases statistical power compared to a cross-sectional design. Furthermore, only longitudinal studies allow for a reliable evaluation of interventions such as treatment effects. Most importantly, only longitudinal measures allow for monitoring the individual patient. In neuroimaging, preprocessing tools are commonly designed for cross-sectional data so that their use in longitudinal data may not fully exploit the advantages of the longitudinal study design with the risk of an overestimation of statistical power or the need of a higher number of subjects, respectively.

Over the last few decades, many dedicated neuroimage analysis tools have been developed to handle longitudinal data. These methods aim to exploit the expected temporal consistency in longitudinal scans to obtain more sensitive measures of longitudinal changes than is possible by analyzing each time point separately. One class of algorithms is designed to detect changes between two consecutive time points without explicitly *segmenting* each scan. These methods work by subtracting the two images to highlight locations of change (Hajnal et al., 1995, Freeborough and Fox, 1997, Lemieux et al., 1998, Battaglini et al., 2014), or, more generally, by tracking corresponding voxel locations over time using nonlinear registration, and analyzing the estimated spatial deformations (Thirion and Calmon, 1999, Rey et al., 2002, Avants et al., 2007, Holland and Dale, 2011, Elliott et al., 2019). Another class of methods explicitly *segments* each time point in longitudinal scans, enforcing temporal consistency either on the segmentations themselves (Metcalf et al., 1992, Solomon and Sood, 2004, Xue et al., 2005, Xue et al., 2006, Wolz et al., 2010, Dwyer et al., 2014, Wei et al., 2021) or on spatial probabilistic atlases that are used to compute them (Shi et al., 2010, Shi et al., 2010, Prastawa et al., 2012, Aubert-Broche et al., 2013, Iglesias et al., 2016, Tustison et al., 2019). In order to make the various time points comparable on a voxel-based level, these methods typically involve a temporal registration step, computed either prior to (Wolz et al., 2010, Aubert-Broche et al., 2013, Gao et al., 2014, Iglesias et al., 2016, Tustison et al., 2019, Schmidt et al., 2019, Wei et al., 2021) or simultaneously with (Xue et al., 2005, Xue et al., 2006, Shi et al., 2010, Shi et al., 2010, Li et al., 2010, Wang et al., 2011, Prastawa et al., 2012, Wang et al., 2013) the segmentations.

To date, most methods for analyzing longitudinal scans are designed to compute only very specific outcome variables, such as change in overall brain size (Hajnal et al., 1995, Freeborough and Fox, 1997, Smith et al., 2001, Smith et al., 2002) or global white/gray matter volume (Xue et al., 2005, Xue et al., 2006, Shi et al., 2010, Shi et al., 2010, Prastawa et al., 2012, Gao et al., 2014), cortical thickness (Nakamura et al., 2011, Wang et al., 2011, Wang et al., 2013, Tustison et al., 2019), white matter lesions (Gerig et al., 2000, Solomon and Sood, 2004, Elliott et al., 2013, Schmidt et al., 2019, Birenbaum and Greenspan, 2016, McKinley et al., 2020, Sepahvand et al., 2020, Denner et al., 2020) or individual brain structures such as the hippocampus (Wolz et al., 2010, Iglesias et al., 2016, Wei et al., 2021). To the best of our knowledge, the most comprehensive tool for longitudinal analysis of structural brain scans is currently the one distributed with FreeSurfer (Reuter et al., 2012, Fischl, 2012). This tool segments many neuroanatomical structures simultaneously (both volumetric “whole-brain” segmentations and parcellations of the cortical surface), and can readily handle data with more than two time points. However, it is specifically designed for T1-weighted (T1w) scans only – as such it is less well suited for studying populations with white matter lesions and other pathologies that are better visualized using other MRI contrasts (such as T2w or FLAIR). Furthermore, a recent study suggests that, even in T1w images, it may be less sensitive to longitudinal changes than the method we describe here (Sederevičius et al., 2021).

The contribution of this paper is twofold. First, we make publicly available a new method for automatically segmenting dozens of neuroanatomical structures from longitudinal scans, using a model-based approach that can take multi-contrast data as input and that can also segment white matter lesions simultaneously. The method is fully adaptive to different MRI contrasts and scanners, and does not put any constraints on the number or the timing of longitudinal follow-up scans.

Second, we conduct an extensive validation of the proposed tool using over 4,500 brain scans acquired with different scanners, field strengths and acquisition protocols, involving both controls and patients suffering from MS and AD. We demonstrate experimentally that the method produces more reliable segmentations in scan-rescan settings than the longitudinal tool in FreeSurfer and than a cross-sectional version of the method, while also being more sensitive to differences in longitudinal changes between patient groups.

**Fig. 1:**
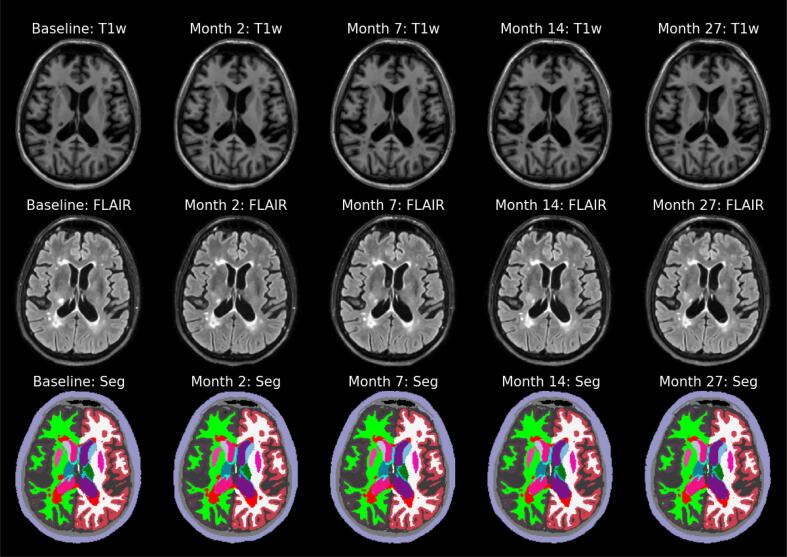
Whole-brain and white matter lesion segmentations (labeled in red) produced by the proposed method from T1w and FLAIR longitudinal scans of an MS patient. T1w=T1-weighted, FLAIR=FLuid Attenuation Inversion Recovery, MS=Multiple Sclerosis.

An example of longitudinal segmentations of an MS patient produced by the proposed method is shown in Fig. 1. A preliminary version of this work, with a limited validation, appeared earlier as a workshop paper (Cerri et al., 2020).

## 2 Existing cross-sectional method – SAMSEG

We build upon a previously validated cross-sectional method for whole-brain segmentation called Sequence Adaptive Multimodal SEGmentation (SAMSEG) (Puonti et al., 2016). SAMSEG segments 41 anatomical structures from brain MRI, and it is fully adaptive to different MRI contrasts and scanners. We here briefly describe the method as we extend it for longitudinal scans in the remainder of the paper.

**Fig. 2:**
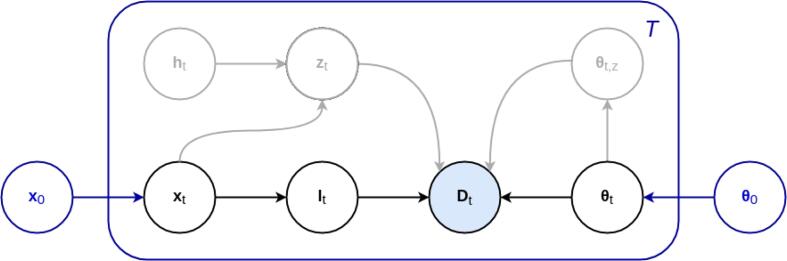
Graphical representation of the proposed longitudinal generative model. For each time point *t*, in black the cross-sectional model of (Puonti et al., 2016), in which image intensities $D_{t}$ are generated from likelihood parameters $\theta_{t}$ and segmentation labels $l_{t}$ – which in turn have been generated from an atlas-based segmentation prior with node positions $x_{t}$. In blue, the proposed additional subject-specific latent variables that encourage temporal consistency between longitudinal scans in the segmentation prior (through $x_{0}$) and in the likelihood function (through $\theta_{0}$). Also shown, in gray, is the cross-sectional lesion extension of (Cerri et al., 2021). For each time point $t,z_{t}$ is a binary white matter lesion segmentation, $h_{t}$ are latent variables encoding lesion shape information, and $\theta_{t,z}$ are lesion intensity parameters constraining lesion appearance. Shading indicates observed variables, while the plate indicates *T* repetition of the included variables.

Let $D=(d_{1},\ldots ,d_{I})$ be the image intensities of a multi contrast scan with *I* voxels, where $d_{i}={\left(d_{i}^{1},\ldots ,d_{i}^{N}\right)}^{T}$ is the vector containing the log-transformed image intensities of voxel *i* for all the available *N* contrasts. Furthermore, let $l={\left(l_{1},\ldots ,l_{I}\right)}^{T}$ be the corresponding segmentation labels, where $l_{i}\in \{1,\ldots ,K\}$ denotes one of the *K* possible anatomical structures assigned to voxel *i*. In order to compute segmentation labels l from image intensities D, we use a generative model illustrated in black in Fig. 2. It defines a forward model composed of two parts: a segmentation prior p(l|x), with parameters x, that encodes spatial information of the labels l, and a likelihood function p(D|l,θ), with parameters θ, that models the imaging process used to obtain the data D. This forward model can be “inverted” to obtain automated segmentations, as detailed below.

### 2.1 Segmentation prior

We use a segmentation prior based on a deformable probabilistic atlas encoded as a tetrahedral mesh (Van Leemput, 2009). The mesh has node positions x, governed by a deformation prior distribution defined as:

(1) $$p\left(x\right)\;\propto \;\exp\left[-K\sum_{m=1}^{M}U_{m}\left(x,\;x_{\mathrm{ref}}\right)\right],$$

where *M* is the number of tetrahedra in the mesh, K>0 controls the stiffness of the mesh, and $U_{m}\left(x,\;x_{\mathrm{ref}}\right)$ is a topology-preserving cost associated with deforming the $m^{\mathrm{th}}$ tetrahedron from its shape in the atlas’s reference position $x_{\mathrm{ref}}$ (Ashburner et al., 2000).

Given a deformed mesh with node positions x, the probability $p(l_{i}=k|x)$ of observing label *k* at voxel *i* is obtained using baricentric interpolation. Assuming conditional independence of the labels between voxels finally yields

$$p\left(l|x\right)=\prod_{i=1}^{I}\;p\left(l_{i}|x\right)\text{.}$$

### 2.2 Likelihood function

We use a multivariate Gaussian intensity model for each of the *K* different structures, and model the bias field artifact as a linear combination of spatially smooth basis functions that is added to the local voxel intensities (Wells et al., 1996, Van Leemput et al., 1999). Letting θ be the collection of the bias field parameters and intensity means and variances, the likelihood function is defined as

$$p\left(D|l,\;\theta \right)=\prod_{i=1}^{I}\;p\left(d_{i}|l_{i},\;\theta \right),$$

$$p(d_{i}|l_{i}=k,\theta )=N(d_{i}|\mu_{k}+C\phi_{i},\Sigma_{k}),$$

$$C=\left(\begin{array}{c} c_{1}^{T}\\ \vdots \\ c_{N}^{T} \end{array}\right),\;c_{n}=\left(\begin{array}{c} c_{n,1}\\ \vdots \\ c_{n,P} \end{array}\right),\;\phi_{i}=\left(\begin{array}{c} \phi_{1}^{i}\\ \vdots \\ \phi_{P}^{i} \end{array}\right),$$

where *P* denotes the number of bias field basis functions, $\phi_{p}^{i}$ is the basis function *p* evaluated at voxel *i*, and $c_{n}$ collects the bias field coefficients for MRI contrast *n*. Furthermore, $\mu_{k}$ and $\Sigma_{k}$ denote the Gaussian mean and variance of structure *k*, respectively. A flat prior is used for the parameters of the likelihood, i.e., p(θ)∝1.

### 2.3 Segmentation

Given an MRI scan D, a corresponding segmentation is obtained by first fitting the model to the data:

(2) $$\hat{x},\hat{\theta }=\underset{x,\theta }{\mathrm{argmax}}\;p\left(x,\theta |D\right)\text{.}$$

The optimization problem in (2) is solved using a coordinate ascent scheme, in which x and then θ are iteratively updated, each in turn. Once the model parameter estimates $\left\{\hat{x},\;\hat{\theta }\right\}$ are available, the corresponding maximum a posteriori (MAP) segmentation is obtained as

$$\hat{l}=\underset{l}{\mathrm{argmax}}\;p\left(l|D,\hat{\theta },\hat{x}\right)\text{.}$$

Since p(l|D,θ^,x^)∝p(D|l,θ^)p(l|x^) and therefore factorizes over *i*, the optimal segmentation label can be computed for each voxel independently:

(3) $${\hat{l}}_{i}=\underset{k}{\mathrm{argmax}}\frac{N\left(d_{i}|{\hat{\mu }}_{k}+\hat{C}\phi_{i},{\hat{\Sigma }}_{k}\right)p\left(l_{i}=k|\hat{x}\right)}{\sum_{k'=1}^{K}N\left(d_{i}|{\hat{\mu }}_{k'}+\hat{C}\phi_{i},{\hat{\Sigma }}_{k'}\right)p\left(l_{i}=k'|\hat{x}\right)}\text{.}$$

More details can be found in (Puonti et al., 2016).

## 3 Longitudinal method – SAMSEG-Long

We now describe how we extend SAMSEG for longitudinal scans. In the remainder of the paper, we call the proposed longitudinal method *SAMSEG-Long*.

In a longitudinal scenario, we aim to compute automatic segmentations ${\left\{l_{t}\right\}}_{t=1}^{T}$ from *T* consecutive scans with image intensities ${\left\{D_{t}\right\}}_{t=1}^{T}$. In contrast to the cross-sectional setting where each image is treated independently, here we can exploit the fact that all images belong to the same subject to produce more consistent (and potentially more accurate) segmentations. Towards this end, we introduce subject-specific latent variables $x_{0}$ and $\theta_{0}$ in the segmentation prior and likelihood function of SAMSEG, respectively. The purpose of these additional components in the model – illustrated in blue in Fig. 2 – is to impose a statistical dependency between the time points, encouraging the segmentations to remain similar to one another.

In the following, we describe how the new subject-specific latent variables are defined in the segmentation prior and likelihood function, and how we obtain the corresponding segmentations accordingly. We will use the notation $x_{t}$ and $\theta_{t}$ to indicate the parameters of the prior and likelihood function at time *t*, respectively.

### 3.1 Segmentation prior

In order to obtain temporal consistency in the segmentation prior, we use the concept of a “subject-specific atlas” (Iglesias et al., 2016): a deformation of the cross-sectional atlas to represent the average subject-specific anatomy across all time points. In particular, we use

$$p\left({\left\{x_{t}\right\}}_{t=1}^{T}|x_{0}\right)=\overset{T}{\underset{t=1}{\prod \;}}p\left(x_{t}|x_{0}\right)$$

with

$$p\left(x_{t}|x_{0}\right)\;\propto \;\exp\left[-K\sum_{m=1}^{M}U_{m}\left(x_{t},\;x_{0}\right)\right],$$

where $x_{0}$ are latent atlas node positions encoding subject-specific brain shape, with prior

$$p\left(x_{0}\right)\;\propto \;\exp\left[-K_{0}\sum_{m=1}^{M}U_{m}\left(x_{0},\;x_{\mathrm{ref}}\right)\right]\text{.}$$

Here the mesh stiffness $K_{0}$ is a hyperparameter of the model, the value of which we determine empirically using cross-validation (cf. Section 4.1).

Note that this formulation of the longitudinal segmentation prior is very flexible, as it does not impose specific temporal trajectories (e.g., monotonic growth) on the anatomy of the subject. Furthermore, by using a very large value for its hyperparameter $K_{0}$, $x_{0}$ can be forced to remain close to $x_{\mathrm{ref}}$, so that cross-sectional segmentation prior of (1) for each individual time point is retained as a special case.

### 3.2 Likelihood function

In a similar vein, we also introduce subject-specific latent variables to encourage temporal consistency in the Gaussian intensity models. For each anatomical structure *k*, we condition its Gaussian parameters ${\left\{\mu_{t,k},\Sigma_{t,k}\right\}}_{t=1}^{T}$ on latent variables $\left\{\mu_{0,k},\Sigma_{0,k}\right\}$ using a normal-inverse-Wishart (NIW) distribution:

$$p\left({\left\{\theta_{t}\right\}}_{t=1}^{T}|\theta_{0}\right)=\prod_{t=1}^{T}\;p\left(\theta_{t}|\theta_{0}\right)$$

with

$$p\left(\theta_{t}|\theta_{0}\right)\propto \prod_{k=1}^{K}N\left(\mu_{t,k}|\mu_{0,k},P_{0,k}^{-1}\Sigma_{t,k}\right)\mathrm{IW}\left(\Sigma_{t,k}|P_{0,k}\Sigma_{0,k},P_{0,k}-N-2\right),$$

where $\theta_{0}={\left\{\mu_{0,k},\Sigma_{0,k}\right\}}_{k=1}^{K}$ collects the latent variables of all structures, and is assumed to have a flat prior: $p(\theta_{0})\propto 1$. The effect of this longitudinal model is to encourage the means and variances of each structure to remain similar to some “prototype” $\mu_{0,k}$ and $\Sigma_{0,k}$, respectively, without having to specify a priori what values these prototypes should take. The strength of this effect is governed by a hyperparameter $P_{0,k}⩾0$ for each structure, which we determine empirically using cross-validation (cf. Section 4.1).

Note that no temporal regularization is added to the parameters of the bias field model, since the bias field will typically vary between MRI sessions. (Differences in global intensity scaling between time points are automatically included in the bias field model as well.) Furthermore, by choosing hyperparameters $P_{0,k}=0$ the temporal regularization of the Gaussian parameters can be switched off, in which case the proposed likelihood function devolves into that of the cross-sectional SAMSEG method for each time point separately (Section 2.2).

### 3.3 Segmentation

As in the cross-sectional case, segmentations are obtained by first fitting the model to the data:

(4) $${\hat{\theta }}_{0},{\hat{x}}_{0},{\left\{{\hat{x}}_{t},{\hat{\theta }}_{t}\right\}}_{t=1}^{T}=\underset{{}_{\theta_{0},x_{0},{\left\{x_{t},\theta_{t}\right\}}_{t=1}^{T}}}{\mathrm{argmax}}p\left(\theta_{0},x_{0},{\left\{x_{t},\theta_{t}\right\}}_{t=1}^{T}\;|{\left\{D_{t}\right\}}_{t=1}^{T}\right)\text{.}$$

We optimize (4) with a coordinate ascent scheme, where we iteratively update each variable one at the time. Because $p(x_{t}|x_{0})$ is of the same form as the cross-sectional segmentation prior, and the NIW distribution used in $p(\theta_{t}|\theta_{0})$ is the conjugate prior for the mean and variance of a Gaussian distribution, estimating $x_{t}$ and $\theta_{t}$ from $D_{t}$ for given values of $x_{0}$ and $\theta_{0}$ simply involves performing an optimization of the form of (2) for each time point *t* separately. Conversely, for given values ${\left\{x_{t},\theta_{t}\right\}}_{t=1}^{T}$ the update for $\theta_{0}$ is given in closed form:

$$\begin{array}{c} \mu_{0,k\;}\leftarrow {\left(\sum_{t=1}^{T},\Sigma_{t,k}^{-1}\right)}^{-1}\sum_{t=1}^{T}\Sigma_{t,k}^{-1}\mu_{t,k},\\ \Sigma_{0,k}^{-1}\;\leftarrow \left(\frac{1}{T}\sum_{t=1}^{T}\Sigma_{t,k}^{-1}\right)\;\frac{P_{0,k}}{P_{0,k}-N-2}, \end{array}$$

whereas updating $x_{0}$ involves the optimization

$$\underset{x_{0}}{\mathrm{argmin}}\;\sum_{m=1}^{M}\left[K_{0}U_{m}\left(x_{0},\;x_{\mathrm{ref}}\right)+K\sum_{t=1}^{T}U_{m}\left(x_{t},\;x_{0}\right)\right],$$

which we solve numerically using a limited-memory BFGS algorithm.

Once all parameters are estimated, we obtain segmentations as described in the cross-sectional setting, i.e., by using (3) for each time point separately.

### 3.4 Extension for white matter lesion segmentation

We have previously extended the cross-sectional SAMSEG method using an augmented model that allows it to robustly segment lesions in the white matter (Cerri et al., 2021). In this extended version, lesions are modeled with an extra Gaussian in the likelihood function, and with lesion-specific location and shape constraints in the segmentation prior. These additional lesion components are illustrated in gray in the graphical model of Fig. 2; we refer the reader to (Cerri et al., 2021) for an in-depth description.

The SAMSEG version with lesion segmentation ability can easily be integrated into SAMSEG-Long, as illustrated in Fig. 2. Due to the highly varying temporal behavior of white matter lesions, we do not explicitly constrain their shape and appearance over time, as this could potentially degrade the segmentation performance of the method. As a result, there are no direct dependencies between the lesion-specific components of the model and the latent variables $x_{0}$ and $\theta_{0}$ (note the absence of direct arrows between the two sets of variables in Fig. 2). Computing longitudinal whole-brain and white matter lesion segmentations can therefore follow the same procedure described before (cf. Section 3.3), with only a few modifications in how, for each time point *t*, the parameters $x_{t}$ and $\theta_{t}$ are estimated, and individual segmentations $l_{t}$ are computed (Cerri et al., 2021).

## 4 Implementation

In our current implementation, it is assumed that all time points have been registered and resampled to the same image grid prior to segmentation. To avoid introducing spurious biases by not treating all time points in exactly the same way (e.g., by resampling follow-up scans to a baseline scan) (Reuter and Fischl, 2011), for this purpose we use an unbiased within-subject template created with an inverse consistent registration method (Reuter et al., 2012). This template is a robust representation of the average subject anatomy over time, and we use it as an unbiased reference to register all time points to, resulting in resampled images that then form the input to our segmentation algorithm. In case of multi-contrast images, this procedure is performed for one specific contrast (T1w in the experiments used in this paper), and the remaining contrasts (FLAIR in the experiments) are subsequently registered and resampled to the first contrast for each time point individually.

To initialize the proposed algorithm, we first apply the cross-sectional method to the unbiased template, and use the estimated model parameters x^ and θ^ to initialize the corresponding parameters $x_{t}$ and $\theta_{t}$ at each time point *t*. The model fitting procedure of (4), which interleaves updating the latent variables $\left\{x_{0},\;\theta_{0}\right\}$ with updating the parameters ${\left\{x_{t},\;\theta_{t}\right\}}_{t=1}^{T}$, is then run for five iterations, which we have found to be sufficient to reach convergence.

**Fig. 3:**
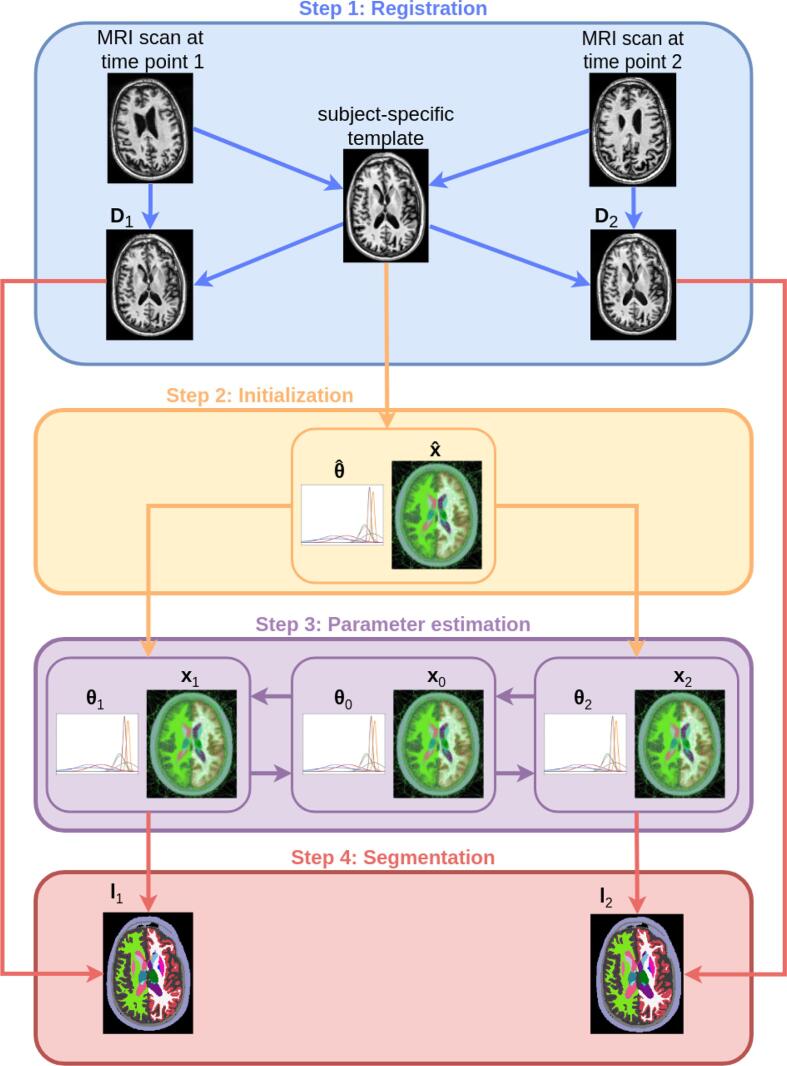
Schematic illustration of the entire longitudinal segmentation process. Although the method is more general, here only the case of single-contrast scans and T=2 time points is shown to avoid cluttering. In Step 1, an unbiased subject-specific template is created with an inverse consistent registration method (Reuter et al., 2012), and each input scan is subsequently registered to it (Section 4). The cross-sectional method (Section 2) is then applied to the template in Step 2, and the estimated parameters x^ and θ^ are used to initialize the corresponding parameters $x_{t}$ and $\theta_{t}$ at each time point *t*. In Step 3, model parameters are estimated in an iterative process (Section 3.3); this involves alternating updates for subject-specific latent variables $x_{0}$ and $\theta_{0}$, with updates for time point parameters ${\left\{x_{t},\theta_{t}\right\}}_{t=1}^{T}$. Once model parameters estimates are available, in Step 4 each time point is segmented accordingly (Eq. (3)).

An illustration of the entire longitudinal segmentation process is provided in Fig. 3. Our implementation builds upon the C++ and Python code of (Puonti et al., 2016, Cerri et al., 2021), and is publicly available from FreeSurfer^1^. Segmenting one subject with 1 mm^3^ isotropic resolution and image size of $256^{3}$ takes approximately 10 min per time point for SAMSEG-Long, while 5 additional minutes per time point are needed when segmenting also white matter lesions (measured on an Intel 12-core i7-8700 K processor).

### 4.1 Hyperparameter tuning

SAMSEG-Long has hyperparameters $K_{0}$ and $P_{0,k}$ that control, respectively, the strength of the regularization in the segmentation prior and likelihood function. Good choices for the value of these hyperparameters will aim to minimize differences between scans acquired within a short interval of time, while simultaneously maximizing the ability to detect known atrophy trajectories in different patient groups.

We therefore tuned these hyperparameters by applying a grid search using 80 test–retest scans and 80 longitudinal scans of both cognitively normal (CN) and AD subjects. In particular, using T1w images from the MIRIAD-TR-HT (CN = 10, AD = 30) and the ADNI-HT (CN = 37, AD = 53) datasets summarized in Table 1 and detailed in Appendix A, we searched from the following values of the hyperparameters: $K_{0}=\{5K,10K,14K,15K,20K\}$ and $P_{0,k}=\{0.25N_{k},0.5N_{k},0.75N_{k},N_{k},1.25N_{k}\}$, where $N_{k}$ is the number of voxels assigned to class *k* in the cross-sectional segmentation of the within-subject template.

**Table 1:** Summary of the experiments and datasets used in the paper. CN=Cognitive Normal, CV=Converted, AD=Alzheimer’s Disease, MS=Multiple Sclerosis, S-MS=Stable-MS, P-MS=Progressive-MS, # scans=total number of scans, tp=time points, avg-tp=average number of time points per subject, time-tp=average time in days between each time point, HT=Hyperparameter Tuning, TR=Test–Retest, IR-FSPGR=Inversion Recovery prepared - Fast SPoiled Gradient Recalled, MP-RAGE=Magnetization Prepared - RApid Gradient Echo, FLAIR=FLuid Attenuation Inversion Recovery. For more details about each individual dataset, see Appendix A.

Experiment	Dataset	Subjects	# scans	avg-tp (min, max)	time-tp (min, max)	Scanner	Sequence
Hyperparameter tuning	MIRIAD-TR-HT	CN=10, AD=30	80	2 (2, 2)	0 (0, 0)	GE Signa 1.5T	IR-FSPGR
	ADNI-HT	CN=37, AD=53	285	3.56 (2,5)	313 (107, 1121)	Multiple 3T scanners	MP-RAGE IR-FSPGR

Test–retest reliability	MIRIAD-TR	CN=13, AD=16	146	2 (2, 2)	0 (0, 0)	GE Signa 1.5T	IR-FSPGR
	OASIS-TR	CN=72, CV=14, AD=64	1845	3.55 (3, 4)	0 (0, 0)	Siemens Vision 1.5T	MP-RAGE
	Munich-TR	MS=2	34	5.67 (5, 6)	3 (2, 7)	Philips Achieva 3T Siemens Verio 3T GE Signa MR750 3T	IR-FSPGR MP-RAGE FLAIR

Detecting disease effects	ADNI	CN=66, AD=64	477	3.70 (2, 5)	298 (65, 903)	Multiple 1.5T and 3T scanners	IR-FSPGR MP-RAGE
	OASIS	CN=72, AD=64	336	2.47 (2, 5)	702 (182, 1510)	Siemens Vision 1.5T	MP-RAGE
	Munich	MS=200 (S-MS=100, P-MS=100)	1289	6.45 (2, 24)	353 (18, 3287)	Philips Achieva 3T	MP-RAGE FLAIR

Longitudinal lesion segmentation	ISBI	MS=14	61	4.36 (4, 6)	391 (299, 503)	Philips 3T	MP-RAGE FLAIR

**Fig. 4:**
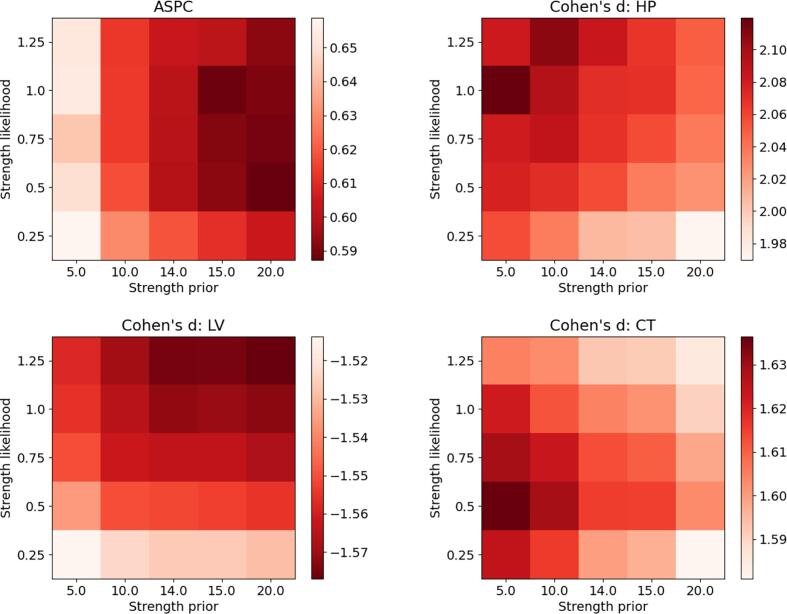
Hyperparameter tuning by grid search over the hyperparameters of the proposed method. Top left: ASPC values across subjects and structures for the 80 test–retest T1w scans of the 40 subjects of the MIRIAD-TR-HT dataset (CN=10, AD=30). Top right and bottom row: Cohen’s effect sizes computed from APCs estimates of the 80 subjects of the ADNI-HT dataset (CN=37, AD=53) for Hippocampus (HP), Lateral Ventricles (LV) and Cerebral Cortex (CT). APSC=Absolute Symmetrized Percent Change, CN=Cognitive Normal, AD=Alzheimer’s Disease.

The results are summarized in Fig. 4, which shows ASPC values in the test–retest scenario, as well as Cohen’s d effect sizes of APC values between CN and AD patients for hippocampus, lateral ventricles and cerebral cortex – three structures known to be strongly affected in AD (Lombardi et al., 2020). The ASPC and APC metrics are defined in detail in Section 5.2, but in short assess volumetric changes between test and retest scans (ASPC), and the yearly rate of volume changes in longitudinal scans (APC), both expressed as a percentage.

The proposed method yielded consistent performance overall, with only minor differences between the various hyperparameter value combinations: ASPC and Cohen’s d values varied within a 5.8% and 3.7% range compared to the average performance, respectively. Nevertheless, for the purpose of having fixed values for these hyperparameters, we used the combination $K_{0}=20K$ and $P_{0,k}=0.5N_{k},\forall k$ for all the experiments described below.

## 5 Experiments

In order to evaluate the performance of SAMSEG-Long, we conducted experiments on multiple datasets acquired with many different scanner platforms, field strengths, acquisition protocols, and image resolutions. These datasets contain images of cognitively normal subjects as well as AD and MS patients, and differ both in the number and timing of their longitudinal follow up scans, as well as in the number of MRI contrasts that are acquired. A summary of the datasets can be found in Table 1, with more detailed information for each individual dataset in Appendix A. Taken together, we believe these datasets are an excellent source to demonstrate the robustness and generalizability of the proposed longitudinal method, which does not need to be retrained or tuned on any of these datasets.

As a first experiment, we evaluated the method’s test–retest reliability. Since test–retest scans are acquired within a minimal interval of time (usually within the same scan session or within a couple of weeks), no biological variations in the various structures are expected, and we therefore evaluated the ability of the method to produce consistent segmentations in these settings. Although this property is essential in a longitudinal segmentation method, an algorithm that produces the same segmentation for each given time point would, by definition, also have perfect test–retest reliability. Additional experiments are therefore needed to evaluate the ability of the method to also detect real longitudinal changes if they exist. Ideally, this would involve comparing the longitudinal segmentations computed by the proposed method with manually delineated longitudinal data. However, to the best of our knowledge, such ground truth data is not currently available. We therefore performed an *indirect* evaluation of the sensitivity of the method, by assessing its ability to detect known differences between the temporal trajectories in different patient groups (CN vs. AD, stable vs. progressive MS).

For all our experiments, we report the performance of the “vanilla” SAMSEG-Long method as described in Section 3.3 – except for MS patients, for which we use the method with its white matter lesion segmentation extension (Section 3.4).

### 5.1 Benchmark methods

In order to benchmark the longitudinal whole-brain segmentation performance of SAMSEG-Long, we compared it against that of SAMSEG (which is cross-sectional), and the longitudinal stream of FreeSurfer 7.2 (Reuter et al., 2012), called Aseg-Long in the remainder of the paper. Aseg-Long is the only publicly available and extensively validated longitudinal method that segments the same neuroanatomical structures as our method, representing a natural benchmark for evaluating its whole-brain segmentation performance. However, other tools exist that have reported better performance for estimating longitudinal volume changes in specific structures, such as the hippocampus, lateral ventricles or gray matter (Nakamura et al., 2014, Guizard et al., 2015). Note that Aseg-Long is unable to process multi-contrast scans, hence no comparison was performed on such data.

For evaluating the lesion segmentation component of SAMSEG-Long, we compared its performance against that of both SAMSEG and the longitudinal white matter lesion segmentation method of (Schmidt et al., 2019), called LST-Long in the remainder of the paper. LST-Long is one the few methods that have a publicly available implementation. It segments lesions from T1-weighted and FLAIR MRI scans with multiple time points, and does not require retraining when tested on unseen data. Unlike our method, however, it does not provide further segmentations of the various neuroanatomical structures beyond white matter lesions.

### 5.2 Metrics and measures

Although the proposed method segments more structures, we concentrated on the following 25 main neuroanatomical regions: left and right cerebral white matter (WM), cerebellum white matter (CWM), cerebellum cortex (CCT), cerebral cortex (CT), lateral ventricle (LV), hippocampus (HP), thalamus (TH), putamen (PU), pallidum (PA), caudate (CA), amygdala (AM), nucleus accumbens (AC) and brain stem (BS). To avoid cluttering, we merged the results between right and left structures. For experiments that include MS patients, white matter lesion (LES) results are also reported.

For evaluating test–retest reliability, we computed the Absolute Symmetrized Percent Change (ASPC) for each structure, defined as

$$\mathrm{ASPC}=\frac{100\cdot |v_{2}-v_{1}|}{(v_{1}+v_{2})/2},$$

where $v_{1}$ and $v_{2}$ represent the volume of the structure in scan 1 and scan 2, respectively. To check for under- and over-segmentation trends, we also computed Symmetrized Percent Change (SPC), defined in the same way as ASPC but without the absolute value.

For assessing a method’s ability to detect disease effects, we computed the Annualized Percentage Change (APC): We fitted a line to the subject’s volumetric measurements, plotted as a function of time from the baseline, and computed the APC as the ratio of its slope to its intercept (evaluated at the time of the first scan). A negative APC value thus corresponds to a yearly shrinkage, in percentage, of the structure of interest, while a positive APC value indicates a yearly growth. Effect sizes between APCs of two patient groups were then computed using Cohen’s d (Cohen, 2013). We also report the minimum number of subjects needed to detect a statistically significant difference in atrophy rates between two patient groups, by first fitting a generalized linear model with APCs and corresponding patient groups. We then performed a power analysis using the computed model coefficients and noise variance, with 80% power and 0.05 significance level (Cohen, 2013).

For evaluating sensitivity to lesion activity in MS patients, we additionally assessed the apparent speed of lesion volume growth and shrinkage between segmentations at consecutive time points. Specifically, we computed annualized lesion volume *increase* (LES_I) and *decrease* (LES_D) as

$$\mathrm{LES}\_I=\frac{1}{T-1}\overset{T-1}{\underset{t=1}{\sum }}\frac{{\mathrm{Nz}}_{t,t+1}}{\Delta_{t,t+1}}$$

and

$$\mathrm{LES}\_D=\frac{1}{T-1}\overset{T-1}{\underset{t=1}{\sum }}\frac{{\mathrm{Nz}}_{t+1,t}}{\Delta_{t,t+1}},$$

respectively. Here ${\mathrm{Nz}}_{t,t+1}$ counts the number of voxels that were labeled as lesion at time point t+1 but not at time point *t*, while $\Delta_{t,t+1}$ is the time passed between scan *t* and scan t+1. Intuitively, LES_I (LES_D) counts by how many voxels the total lesion volume has grown (shrunk) in a year, on average, *ignoring potential lesion areas where there was simultaneous shrinkage (growth)*. In MS, lesions as depicted by MRI not only appear and grow (Gaitán et al., 2011) but also shrink and disappear (Sethi et al., 2017, Battaglini et al., 2022), and these metrics have therefore been suggested as markers of disease activity (Pongratz et al., 2019).

To evaluate the lesion segmentation performance of the proposed method against ground truth delineations, we computed Dice coefficients as:

$${\mathrm{DICE}}_{X,Y}=\frac{2|X\cap Y|}{\left|X|+|Y\right|},$$

where *X* and *Y* denote segmentation masks, and |·| counts the number of voxels in a mask.

## 6 Results

Throughout this section, we will refer to specific datasets with their names as defined in Table 1. As a mnemonic, datasets used to evaluate *test–retest* reliability have an affix “-TR” in their names. Whenever boxpots are used, the median is indicated by a horizontal line, plotted inside boxes that extend from the first to the third quartile values of the data. The range of the data is indicated by whiskers extending from the boxes, with outliers represented by “x” symbols.

### 6.1 Test–retest reliability

**Fig. 5:**
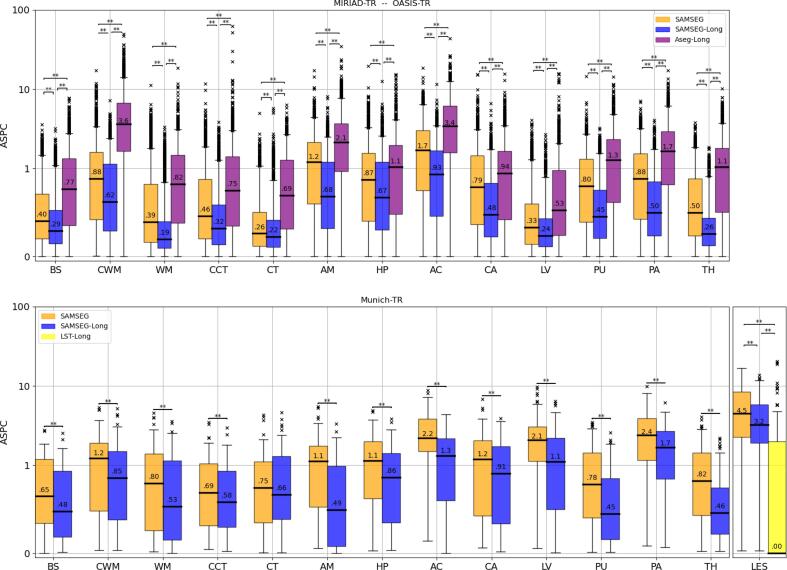
ASPC values for the test–retest T1w scans of the combined MIRIAD-TR (# scans: 146, AD=13, CN=16) and OASIS-TR (# scans: 1845, AD=72, Converted=14, CN=64) datasets (top) and for the test–retest T1w-FLAIR scans of the Munich-TR (# scans: 34, MS=2) dataset (bottom). The yellow boxplot shows the result for LST-Long for white matter lesions. ASPC values were computed for different brain structures and white matter lesion (LES) for the proposed method and the benchmark methods. Within each boxplot, the median ASPC value is also reported. Statistically significant differences between two methods were computed with a Wilcoxon signed-rank test, and are indicated by asterisks (“**” indicates p-value < 0.01). ASPC=Absolute Symmetrized Percent Change, CN=Cognitive Normal, AD=Alzheimer’s Disease, T1w=T1-weighted, FLAIR=FLuid Attenuation Inversion Recovery, MS=Multiple Sclerosis, BS=brain stem, CWM=cerebellum white matter, WM=cerebral white matter, CCT=cerebellum cortex, CT=cerebral cortex, AM=amygdala, HP=hippocampus, AC=nucleus accumbens, CA=caudate, LV=lateral ventricle, PU=putamen, PA=pallidum, TH=thalamus, LES=white matter lesion.

In order to evaluate if the proposed longitudinal method produces consistent segmentations over time, we assessed its performance on test–retest scans of three different datasets: 146 T1w test–retest scans of the 29 subjects of the MIRIAD-TR dataset (CN=13, AD=16), 1845 T1w test–retest scans of the 150 subjects of the OASIS-TR dataset (CN=72, Converted=14, AD=64) and 34 T1w and FLAIR test–retest scans of the 2 MS patients of the Munich-TR dataset. We thus computed automated segmentations for SAMSEG-Long and the benchmark methods, and assessed test–retest reliability performance in terms of ASPC values. When more than two test–retest scans were available for a subject, APSC values were computed for each possible combination of test–retest scan pairs. The results are shown in Fig. 5.

**Fig. B.1:**
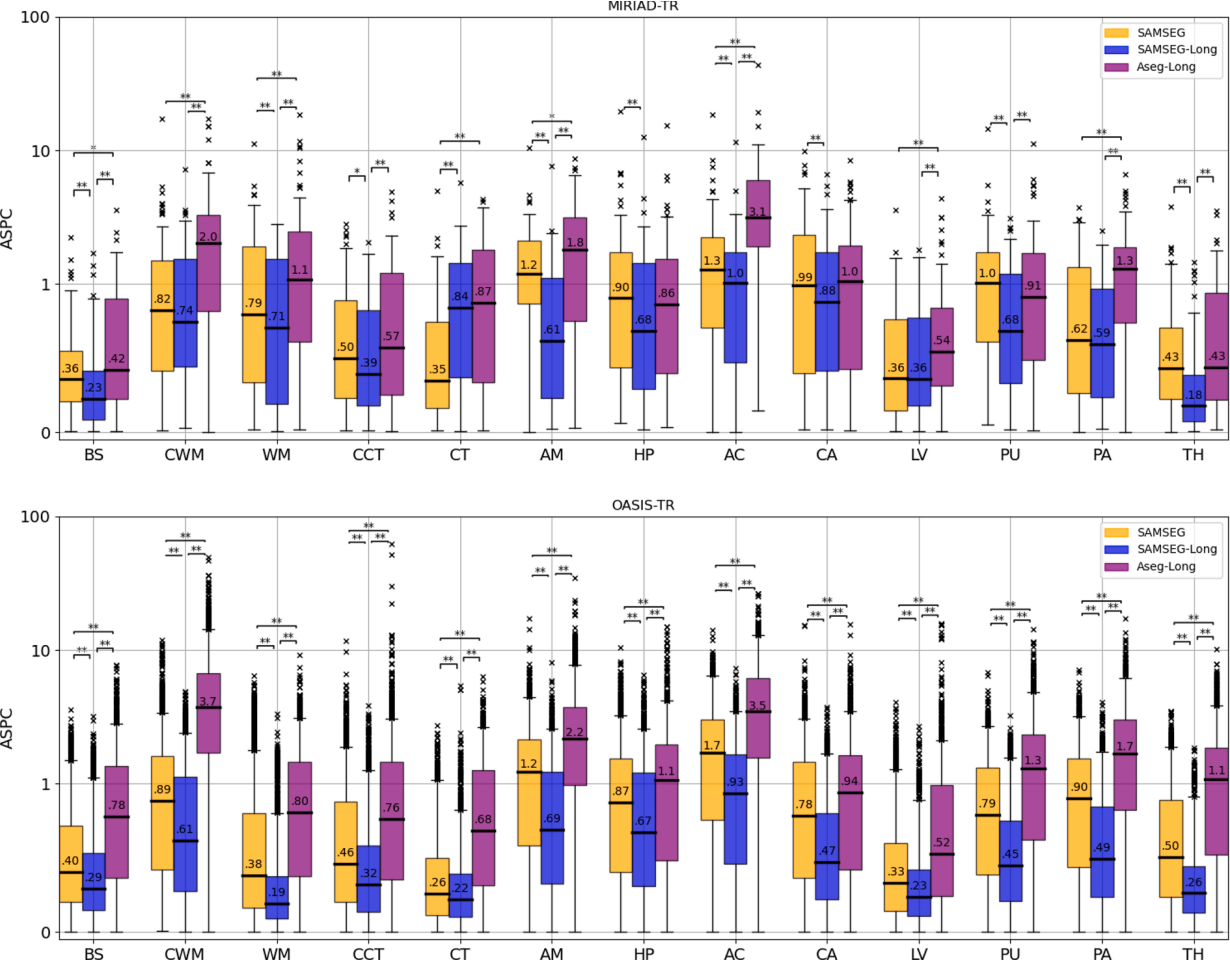
ASPCs for the test–retest T1w scans of the MIRIAD-TR (# scans: 146, AD=13, CN=16) and OASIS-TR (# scans: 1845, AD=72, Converted=14, CN=64) datasets for different brain structures for SAMSEG, SAMSEG-Long and Aseg-Long. Within each boxplot, the median ASPC value is also reported. Statistically significant differences between two methods, were computed with a Wilcoxon signed-rank test, and indicated by asterisks (“**” for p-value < 0.01 and “*” for p-value < 0.05). ASPC=Absolute Symmetrized Percent Change, T1w=T1-weighted, CN=Cognitive Normal, AD=Alzheimer’s Disease, BS=brain stem, CWM=cerebellum white matter, WM=cerebral white matter, CCT=cerebellum cortex, CT=cerebral cortex, AM=amygdala, HP=hippocampus, AC=nucleus accumbens, CA=caudate, LV=lateral ventricle, PU=putamen, PA=pallidum, TH=thalamus.

Since we observed similar results in the MIRIAD-TR and the OASIS-TR datasets, we here only report on their combined results. (We redirect the reader to Fig. B.1 for the results on the individual datasets.) On the combined MIRIAD-TR/OASIS-TR dataset, the median ASPC across all structures was best for SAMSEG-Long: 0.39, compared to 0.62 for SAMSEG and 1.12 for Aseg-Long. Similarly, on the Munich-TR dataset the median ASPC was 0.69 for SAMSEG-Long vs. 1.07 for SAMSEG (Aseg-Long cannot be run on this multi-contrasts dataset). The overall weaker performance on the Munich-TR scans can be explained by the fact that these were acquired within a 3-week interval, whereas the scans of the combined MIRIAD-TR/OASIS-TR dataset were acquired within a single scan session without repositioning. Fig. 5 top shows a high number of outliers for the combined MIRIAD-TR/OASIS-TR dataset, with large ASPC values especially for Aseg-Long. This is mostly due to the high number of test–retest scans (approximately 2,000) of this combined dataset.

As for ASPC values of white matter lesions in the Munich-TR dataset (Fig. 5 bottom right), SAMSEG-Long outperformed SAMSEG (median ASPC: 4.5 vs. 3.2) even though the method does not explicitly regularize white matter lesions longitudinally (see Section 3.4). This may indicate that more consistent model parameter estimates were obtained for SAMSEG-Long compared to SAMSEG as a result of enforcing temporal consistency on all the other structures. We also observe almost perfect test–retest reliability performance for LST-Long (median ASPC: 0.0), thereby outperforming both SAMSEG-LONG and SAMSEG by a large margin in this regard (but see below).

**Fig. B.2:**
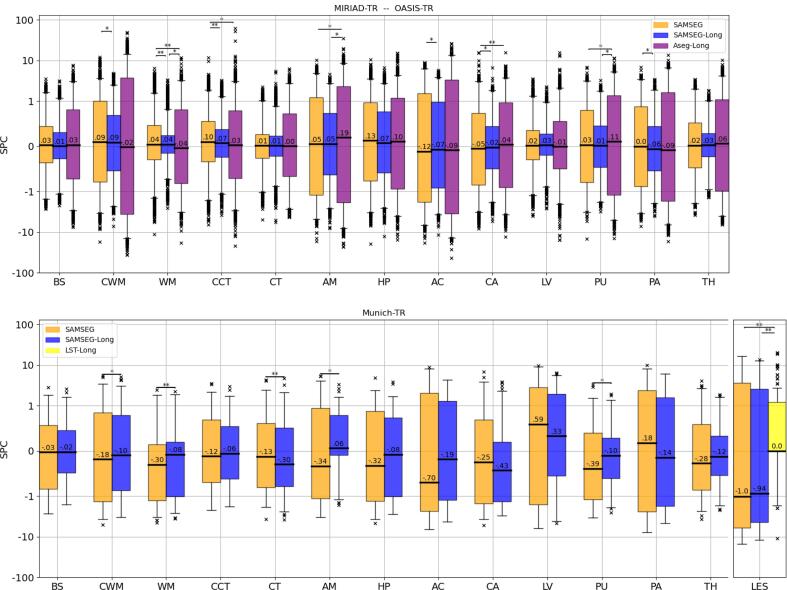
SPC values for the test–retest T1w scans of the combined MIRIAD-TR (# scans: 146, AD=13, CN=16) and OASIS-TR (# scans: 1845, AD=72, Converted=14, CN=64) datasets (top) and for the test–retest T1w-FLAIR scans of the Munich-TR (# scans: 34, MS=2) dataset (bottom). SPCs values were computed for different brain structures and white matter lesion (LES) for the proposed method and the benchmark methods. Within each boxplot, the median SPC value is also reported. Statistically significant differences between two methods were computed with a Wilcoxon signed-rank test, and are indicated by asterisks (“**” for p-value < 0.01, and “*” for p-value < 0.05). SPC=Symmetrized Percent Change, T1w=T1-weighted, FLAIR=FLuid Attenuation Inversion Recovery, AD=Alzheimer’s Disease, CN=Cognitive Normal, MS=Multiple Sclerosis, BS=brain stem, CWM=cerebellum white matter, WM=cerebral white matter, CCT=cerebellum cortex, CT=cerebral cortex, AM=amygdala, HP=hippocampus, AC=nucleus accumbens, CA=caudate, LV=lateral ventricle, PU=putamen, PA=pallidum, TH=thalamus, LES=white matter lesion.

To check whether some of the methods are prone to under- or over-segmenting on test–retest scans, we also report the SPC values (i.e., without taking absolute values) on the same data. The results, shown in Fig. B.2, did not indicate any particular trend in under- or over-segmenting specific structures for any of the methods (median SPC values for all the methods are close to 0), with SAMSEG-Long having the smallest SPC variances, followed by SAMSEG and Aseg-Long.

### 6.2 Detecting disease effects

**Fig. 6:**
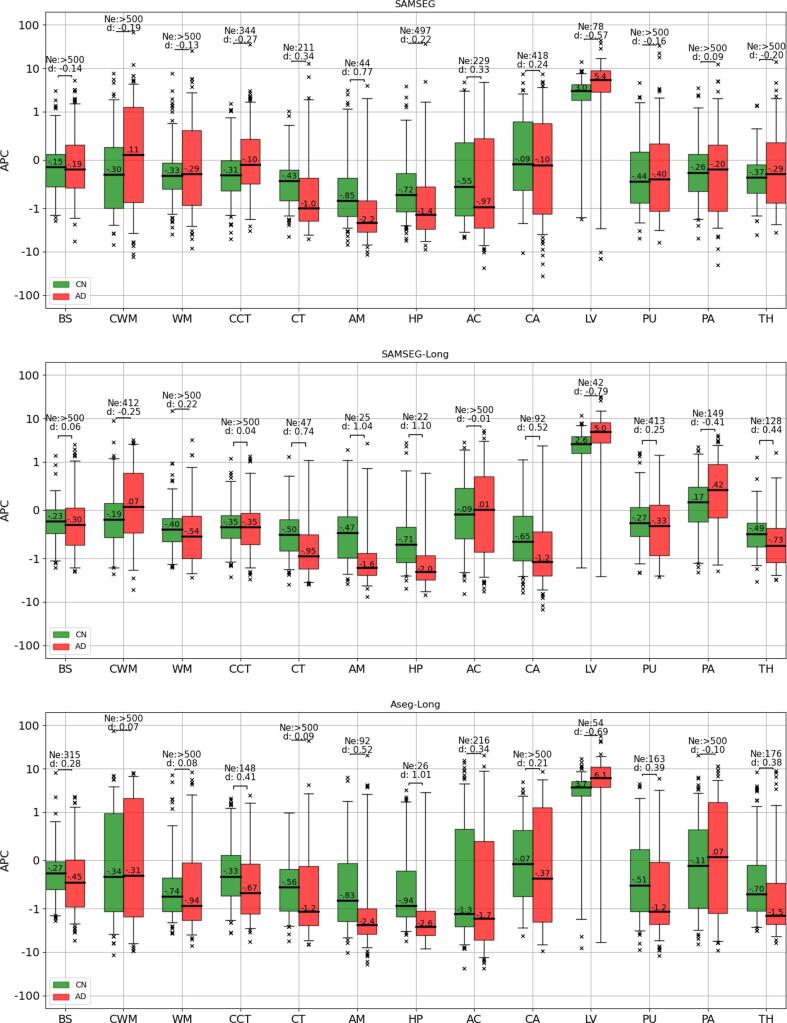
APCs computed from the T1w scans of the 130 subjects of the ADNI dataset (CN=66, AD=64) and 136 subjects of the OASIS dataset (CN=72, AD=64) for SAMSEG, SAMSEG-Long, and Aseg-Long. Cohen’s d effect size (d) and effective number of subjects (Ne) computed from a power analysis (80% power, 0.05 significance level) are reported above each pair of box plots. Within each boxplot, the median APC value is also indicated. APC=Annualized Percentage Change, CN=Cognitive Normal, AD=Alzheimer’s Disease, T1w=T1-weighted, BS=brain stem, CWM=cerebellum white matter, WM=cerebral white matter, CCT=cerebellum cortex, CT=cerebral cortex, AM=amygdala, HP=hippocampus, AC=nucleus accumbens, CA=caudate, LV=lateral ventricle, PU=putamen, PA=pallidum, TH=thalamus.

**Fig. B.3:**
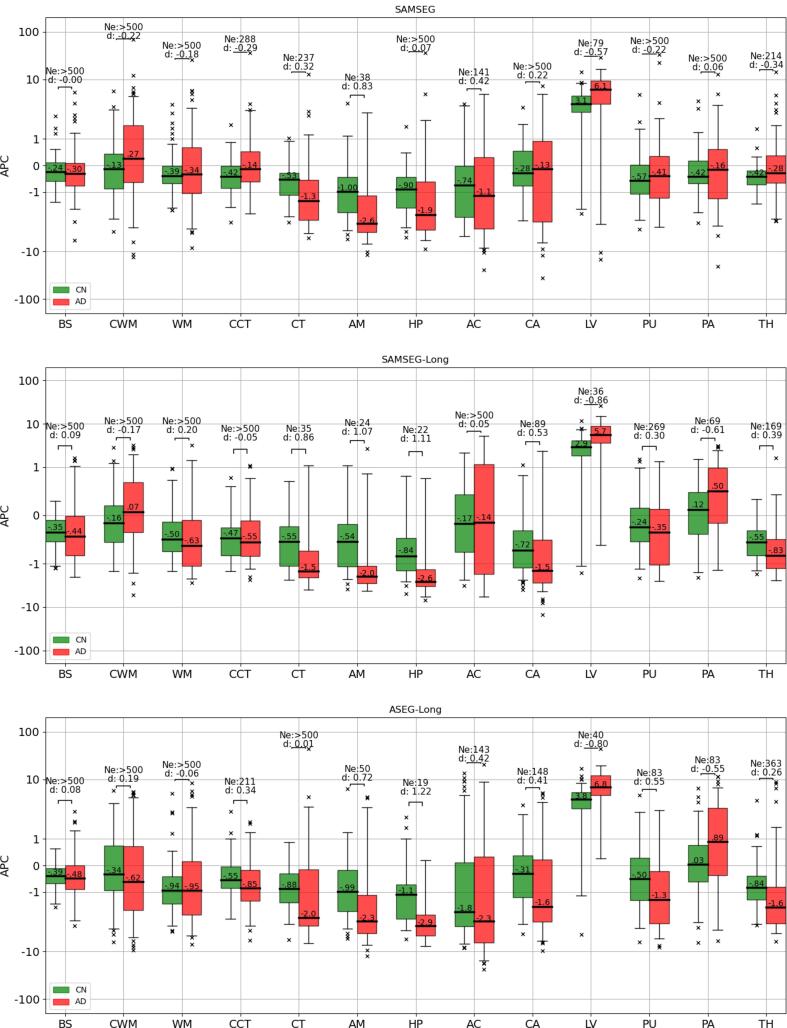
APCs computed from the T1W scans of the 130 subjects of the ADNI dataset (CN=66, AD=64) for SAMSEG, SAMSEG-Long, and Aseg-Long. Cohen’s d effect size (d) and effective number of subjects (Ne) computed from a power analysis (80% power, 0.05 significance level) are reported above each pair of box plots. Within each boxplot, the median APC value is also reported. APC=Annualized Percentage Change, CN=Cognitive Normal, AD=Alzheimer’s Disease, T1w=T1-weighted, BS=brain stem, CWM=cerebellum white matter, WM=cerebral white matter, CCT=cerebellum cortex, CT=cerebral cortex, AM=amygdala, HP=hippocampus, AC=nucleus accumbens, CA=caudate, LV=lateral ventricle, PU=putamen, PA=pallidum, TH=thalamus.

**Fig. B.4:**
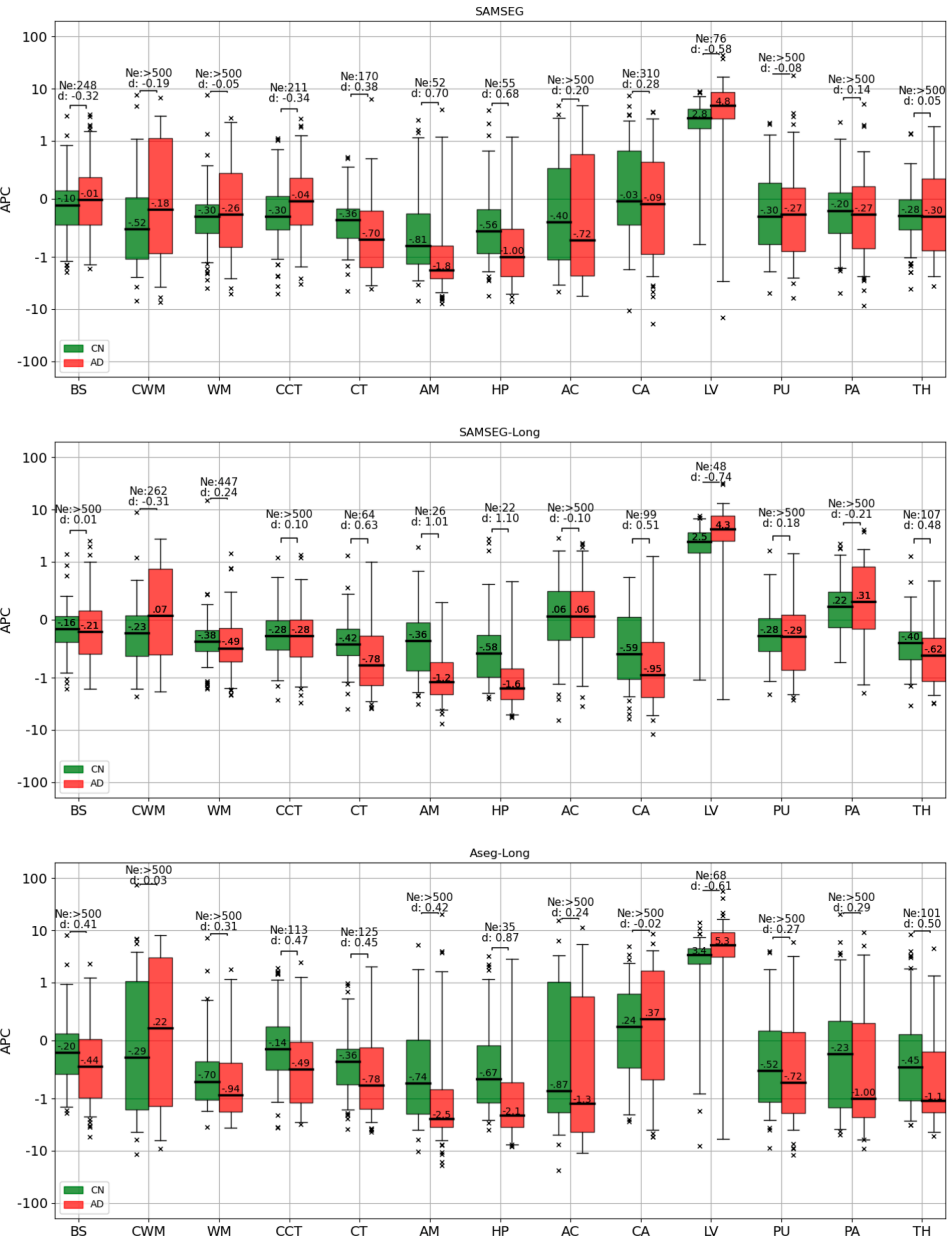
APCs computed from the T1W scans of the 136 subjects of the OASIS dataset (CN=72, AD=64) for SAMSEG, SAMSEG-Long, and Aseg-Long. Cohen’s d effect size (d) and effective number of subjects (Ne) computed from a power analysis (80% power, 0.05 significance level) are reported above each pair of box plots. Within each boxplot, the median APC value is also reported. APC=Annualized Percentage Change, CN=Cognitive Normal, AD=Alzheimer’s Disease, T1w=T1-weighted, BS=brain stem, CWM=cerebellum white matter, WM=cerebral white matter, CCT=cerebellum cortex, CT=cerebral cortex, AM=amygdala, HP=hippocampus, AC=nucleus accumbens, CA=caudate, LV=lateral ventricle, PU=putamen, PA=pallidum, TH=thalamus.

Since we found similar findings across the two datasets, Fig. 6 shows the results for both datasets combined to ease readability. (We redirect the reader to Fig. B.3 and Fig. B.4 for individual dataset results.) All the methods were able to capture well-known differences in the atrophy trajectories of the hippocampus, amygdala and lateral ventricles between CN and AD patients (Lombardi et al., 2020). Effect sizes in these three structures differed between methods, with SAMSEG-Long having higher values for Cohen’s d [0.79–1.10], followed by Aseg-Long [0.52–1.01] and SAMSEG [0.22–0.77]. The results of the power analysis closely mimick these findings: SAMSEG-Long requires fewer subjects to detect the differences in APC [22–42] compared to Aseg-Long [26–54] and SAMSEG [44–497]. Interestingly, only SAMSEG-Long showed a strong effect size for cerebral cortex (Cohen’s d: 0.74), whose atrophy trajectories are known to differ between CN and AD patients (Edmonds et al., 2020), while both Aseg-Long and SAMSEG report lower effect sizes (Aseg-Long: 0.09, SAMSEG: 0.34).

**Fig. 7:**
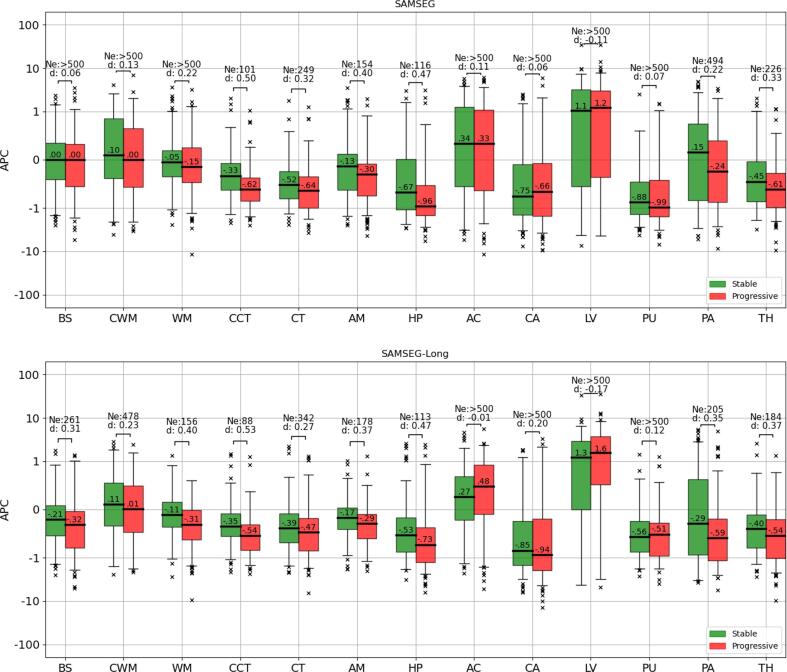
APCs of several structures computed from the T1w and FLAIR scans of the 200 patients of the Munich dataset (100 stable MS, and 100 progressive MS) for SAMSEG and SAMSEG-Long. For each comparison, Cohen’s d effect size (d) and effective number of subjects (Ne) computed from a power analysis (80% power, 0.05 significance level) are shown above the boxplots. Within each boxplot, the median APC value is also reported. APC=Annualized Percentage Change, T1w=T1-weighted, FLAIR=FLuid Attenuation Inversion Recovery, MS=Multiple Sclerosis, BS=brain stem, CWM=cerebellum white matter, WM=cerebral white matter, CCT=cerebellum cortex, CT=cerebral cortex, AM=amygdala, HP=hippocampus, AC=nucleus accumbens, CA=caudate, LV=lateral ventricle, PU=putamen, PA=pallidum, TH=thalamus.

**Fig. 8:**
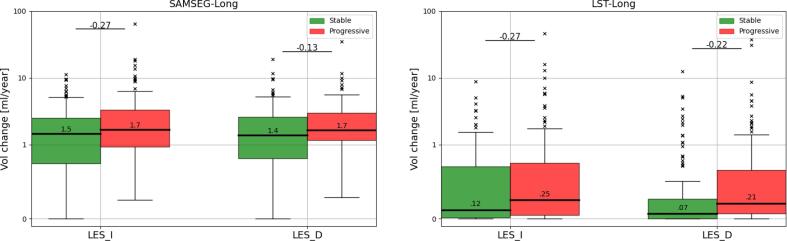
Lesion volume increase (LES_I) and decrease (LES_D) computed from the T1w and FLAIR scans of the 200 patients of the Munich dataset (100 stable MS, and 100 progressive MS) for SAMSEG-Long and LST-Long. For each comparison, Cohen’s d effect size is shown above the boxplots. Within each boxplot, the median value is also reported. T1w=T1-weighted, FLAIR=FLuid Attenuation Inversion Recovery, MS=Multiple Sclerosis.

Fig. 7 shows the same experiment for the 100 stable vs. the 100 progressive MS patients of the Munich dataset. Both SAMSEG-Long and SAMSEG yielded large differences in the atrophy trajectories of the cerebellum cortex, amygdala, hippocampus and thalamus, with SAMSEG-Long yielding nominally higher effect sizes and smaller sample sizes (higher power) compared to SAMSEG in these structures (Cohen’s d: [0.37–0.53 vs. 0.33–0.50], sample sizes: [88–184 vs. 101–226]). These results are in line with previous studies showing more marked atrophy trajectories in progressive MS patients than stable MS patients (Eshaghi et al., 2018, Cagol et al., 2022). We also report in Fig. 8 the yearly volume increase and decrease of lesions – as defined in Section 5.2, i.e., ignoring simultaneous shrinkage and growth, respectively – in stable and progressive MS patients, both for SAMSEG-Long and LST-Long. (Note that we cannot report such results for SAMSEG, as its segmentations are not longitudinally registered, therefore not allowing voxelwise lesion comparisons.) Similar to the findings in (Pongratz et al., 2019), where lesion volume increase and decrease were found to be comparable in size but larger in more active patients, both SAMSEG-Long and LST-Long detected more lesion changes (in both directions, i.e., lesion growth and shrinkage) in the progressive patients compared to the stable ones. Both methods yielded similar effect sizes between the two patient groups, but in absolute terms the lesion volume changes estimated by LST-Long were an order of magnitude smaller than the ones computed by SAMSEG-Long (0.07–0.25 ml/year vs. 1.4–1.7 ml/year). Considering also the almost perfect test–retest performance of LST-Long in Section 6.1, it seems that the method may be over-regularizing over time.

**Fig. 9:**
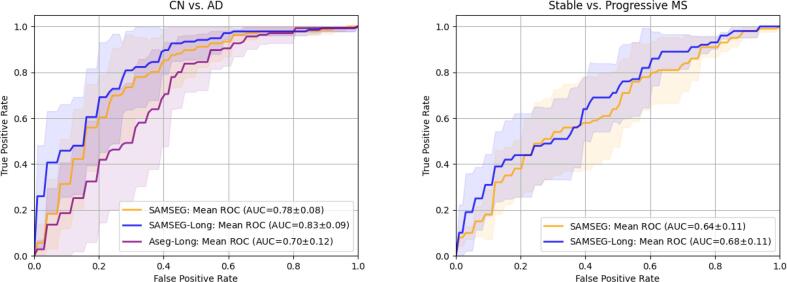
ROC curves for an LDA classifier trained on APC values computed from the T1w scans of the ADNI (CN=66, AD=64) and OASIS (CN=72, AD=64) datasets (left) and from the T1w and FLAIR scans of the Munich dataset (MS stable=100, MS progressive=100) (right). LDA classifiers were trained and tested using a 5-fold cross-validation procedure. For each method, the area under the curve (AUC) is reported, the mean ROC curve is displayed as a solid line, and the shaded area represents ±1 standard deviation error from the mean ROC curve. ROC=receiver operating characteristic curves, LDA=linear discriminant analysis, APC=Annualized Percentage Change, CN=Cognitive Normal, AD=Alzheimer’s Disease, MS=Multiple Sclerosis, T1w=T1-weighted, FLAIR=FLuid Attenuation Inversion Recovery, MS=Multiple Sclerosis.

As a final experiment to compare the various methods’ ability to detect longitudinal disease effects, we assessed whether their APC values contain enough information to correctly classify individual subjects into their respective population groups. In contrast to previous experiments that focused on each structure independently, here we utilized all structures simultaneously: For each method, we trained and tested a linear discriminant analysis (LDA) classifier on APC values using a 5-fold cross-validation procedure. For each fold, 80% of the data was used for training and the remaining 20% for testing. Fig. 9 (left) shows receiver operating characteristic curves (ROC) obtained by training LDA classifiers on APC values computed from the ADNI and OASIS dataset for CN and AD patients. The LDA classifier trained on APC values computed by SAMSEG-Long achieved the highest area under the curve (0.83), followed by the classifiers trained on SAMSEG and Aseg-Long APC values (SAMSEG: 0.78, Aseg-Long: 0.70). Using the same cross-validation procedure, we also trained an LDA classifier on the APC values computed from the Munich dataset for stable and progressive MS patients, and reported ROC curves in Fig. 9 (right). In line with the previous experiment, the LDA classifier trained on SAMSEG-Long APC values obtained a higher area under the curve compared to the classifier trained on SAMSEG APC values (0.68 vs. 0.64).

### 6.3 Longitudinal lesion segmentation performance

In order to directly compare automatic longitudinal lesion segmentations against “ground truth” lesion annotations performed by human experts, we analyzed the 14 MS patients with longitudinal scans provided by the ISBI dataset. Since the heavy preprocessing applied to this data proved problematic for LST-Long, we were unable to get results with this method, and therefore only report performance for SAMSEG and SAMSEG-Long.

**Fig. 10:**
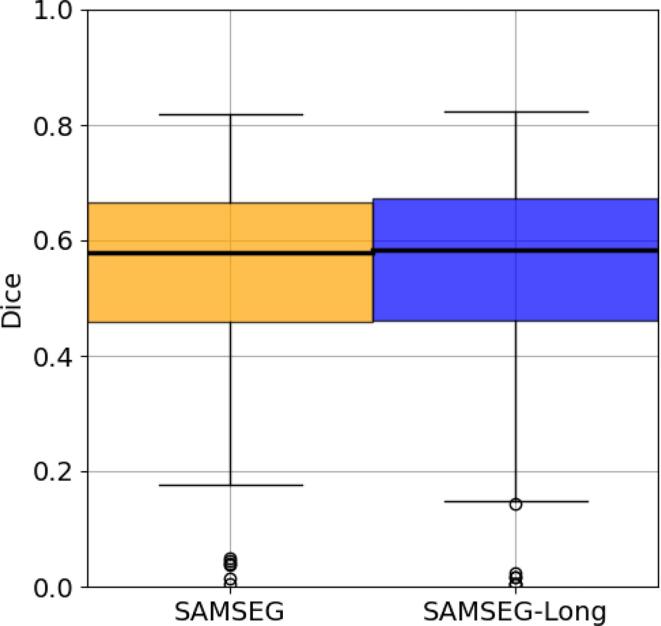
Longitudinal lesion segmentation performance in terms of Dice overlaps with manual raters computed from T1w and FLAIR scans on the 14 MS subjects of the ISBI dataset for SAMSEG and SAMSEG-Long. Each automatic segmentation is compared against each of the two manual segmentations provided in the dataset. No statistically significant difference was detected between the two methods using a two-tailed paired t-test. T1w=T1-weighted, FLAIR=FLuid Attenuation Inversion Recovery, MS=Multiple Sclerosis.

The ISBI challenge website^2^ allows users to upload lesion segmentation masks, and ranks submissions according to an overall lesion segmentation performance score that takes into account Dice overlap, volume correlation, surface distance, and a few other metrics against manual annotations that remain hidden (see (Carass et al., 2017) for details). A score of 100 indicates perfect correspondence, while 90 is meant to correspond to human inter-rater performance (Carass et al., 2017, Styner et al., 2008). SAMSEG obtained a score of 88.31, while SAMSEG-Long similarly scored 88.61. The Dice coefficients between the manual and the corresponding automatic lesion segmentations – computed for each rater, subject and time point individually – are also provided by the website, and are summarized in Fig. 10. The median Dice score was around 0.58 for both SAMSEG and SAMSEG-Long, and no statistical significance was found between the two methods.

When interpreting these results, it is worth pointing out that, although the ISBI data itself is longitudinal, the *manual annotations* are not: As detailed in (Carass et al., 2017), the human raters were presented with the scan of each time point independently, resulting in poor longitudinal consistency in their manual annotations. Furthermore, both SAMSEG and SAMSEG-Long were used “out of the box” without any form of additional tuning. This should be taken into account when comparing their numerical scores against those obtained by methods that were specifically optimized for the exact scanner and imaging protocol of the challenge (typically by training on the matched training data that is also provided by the challenge).

## 7 Discussion and conclusion

In this paper we have proposed and evaluated a new method for segmenting dozens of neuroanatomical structures from longitudinal MRI scans. Temporal regularization is achieved by introducing a set of subject-specific latent variables in an existing cross-sectional segmentation method. An extension for segmenting white matter lesions is also available, allowing users to simultaneously track lesion evolution and morphological changes in various brain structures in e.g., patients suffering from MS. The proposed method does not make any assumptions on the scanner, the MRI protocol, or the number and timing of longitudinal follow-up scans, and is publicly available as part of the open-source neuroimaging package FreeSurfer.

Our experiments indicate that the proposed method has better test–retest reliability compared to benchmark methods, and that it is more sensitive to disease-related changes in AD and MS. In other words, our new tool generated results that were both more sensitive and more specific – suggesting that its use may bring several advantages, such as the need for fewer subjects in longitudinal studies, a better stratification of patients, and more precise evaluation of treatment efficacy.

The robustness and generalizability of the method across different scanner platforms, field strengths, acquisition protocols and image resolutions was demonstrated by its successful “out-of-the-box” application (i.e., without any form of retraining or retuning) on a diverse set of longitudinal datasets. These datasets included single- and multi-contrast longitudinal scans with a range of time gaps and total number of time points, from both healthy and diseased subjects, comprising over 4,500 MRIs in total. The cross-sectional methods the proposed technique builds upon have themselves previously been validated, by comparing their segmentations against those of manual raters on images acquired with different scanners and imaging protocols (Puonti et al., 2016, Cerri et al., 2021). Although this provides evidence of the generalizability of the method, a direct evaluation of its actual *longitudinal* segmentation performance has been hampered by the lack of manual longitudinal annotations that could serve as “ground truth”. As is common in the longitudinal segmentation literature, we therefore resorted to an *indirect* validation and tested the ability of the method to detect disease-related temporal changes instead. This, however, has the limitation that higher sensitivity to group differences does not necessarily imply anatomically more correct segmentations.

Although we believe the proposed tool will be helpful for researchers and clinicians investigating temporal-morphological changes in the brain, the method still has several limitations. First, the method currently only produces volumetric whole-brain segmentations, as opposed to the longitudinal tool of (Reuter et al., 2012) that additionally also computes detailed longitudinal parcellations of the cortical surface. Second, although our tool can segment white matter lesions from longitudinal scans acquired with conventional MRI sequences, the signal changes that it detects in such images are nonspecific, with several different processes all resulting in similar MRI intensity profiles (Sethi et al., 2017, Pongratz et al., 2019). Disentangling the various underlying pathological changes due to e.g., demyelination, remyelination, inflammation or edema may ultimately become feasible with more advanced MRI techniques (Pirko and Johnson, 2008, Oh et al., 2019), but will likely require further development of the image analysis techniques described here. Third, the method does not currently exploit the time dimension explicitly to constrain (Metcalf et al., 1992, Welti et al., 2001, Solomon and Sood, 2004) or analyze lesion evolution (Thirion and Calmon, 1999, Gerig et al., 2000, Rey et al., 2002, Bosc et al., 2003, Elliott et al., 2013) in detail. Dedicated methods for detecting *new* or *growing* lesions by comparing two consecutive time points, in particular, have received ongoing attention (Commowick et al., 2021, Diaz-Hurtado et al., 2022): Many methods rely on image subtraction techniques (Sweeney et al., 2013, Battaglini et al., 2014, Ganiler et al., 2014, McKinley et al., 2020, Sepahvand et al., 2020, Krüger et al., 2020, Klistorner et al., 2021), while others use spatial deformation information (Elliott et al., 2019, Preziosa et al., 2022). Although this type of functionality is not directly provided by the proposed tool, its ability to tightly standardize longitudinal images – both in terms of removing global intensity scaling differences and bias field artifacts, and in terms of establishing accurate longitudinal nonlinear registrations across the various time points – may be leveraged to further develop such methods.

## CRediT authorship contribution statement

**Stefano Cerri:** Conceptualization, Methodology, Software, Formal analysis, Validation, Visualization, Writing - original draft. **Douglas N. Greve:** Software, Writing - review & editing. **Andrew Hoopes:** Software, Writing - review & editing. **Henrik Lundell:** Resources, Writing - review & editing. **Hartwig R. Siebner:** Resources, Writing - review & editing. **Mark Mühlau:** Resources, Writing - review & editing, Funding acquisition. **Koen Van Leemput:** Supervision, Conceptualization, Methodology, Software, Writing - review & editing, Funding acquisition.

## Declaration of Competing Interest

The authors declare that they have no known competing financial interests or personal relationships that could have appeared to influence the work reported in this paper.

## Data Availability

Data will be made available on request.

## Appendix A Datasets details

We use disparate datasets for validating the proposed longitudinal method against benchmark methods, as well as for tuning the hyperparameters of the model. We here describe them in detail:

• **MIRIAD-TR-HT** (Malone et al., 2013): This dataset consists of test–retest scans of 10 healthy elderly people and 30 AD patients from the Minimal Interval Resonance Imaging in Alzheimer’s Disease (MIRIAD) project^3^. T1-weighted (T1w) scans with voxel size of 0.9375×0.9375×1.5 mm were acquired on a GE Signa 1.5T scanner using an Inversion Recovery prepared - Fast SPoiled Gradient Recalled (IR-FSPGR) sequence. For each subject, 2 test–retest images were acquired without removing the patient from the scanner, for a total of 80 scans.
• **ADNI-HT**: This dataset consists of T1w longitudinal scans of 80 subjects from the ADNI project^4^. Different 3T scanners from multiple sites were used for scanning subjects. For each subject, T1w scans were acquired using an IR-FSPGR or MP-RAGE sequence at different image resolution ([min–max]: [1.18–1.21] × [0.92–1.31] × [0.93–1.29] mm), with minor resolution variations between follow up scans (⩽0.01 mm in each axis). Subjects were scanned on average 3.56 times (minimum: 2 times; maximum: 5 times; 285 scans in total), with a mean interval between scans equal to 313 days (minimum: 107 days; maximum: 1121 days). The mean age at baseline was 75 years (minimum: 57; maximum: 90). Subjects were divided into two groups: cognitive normal (CN) (n=37) and AD (n=53).
• **MIRIAD-TR** (Malone et al., 2013): This dataset consists of test–retest scans of 13 healthy elderly people and 16 AD patients from the MIRIAD project – distinct from the subjects of the MIRIAD-TR-HT dataset. For each subject, 2 test–retest images were acquired at 2 or 3 different times without removing the patient from the scanner, for a total of 146 scans.
• **ADNI**: This dataset consists of T1w longitudinal scans of 130 subjects from the ADNI project – distinct from the subjects of the ADNI-HT dataset. Different scanners from multiple sites and multiple field strengths (1.5T and 3T) were used for scanning subjects. For each subject, T1w scans were acquired using an IR-FSPGR or MP-RAGE sequence at different image resolutions ([min–max]: [1.17–1.21] × [0.91–1.31] × [0.92–1.31] mm), with minor resolution variations between follow-up scans (⩽0.01 mm in each axis). Subjects were scanned on average 3.70 times (minimum: 2 times; maximum: 5 times; 477 scans in total), with a mean interval between scans equal to 298 days (minimum: 65 days; maximum: 903 days). The mean age at baseline was 76 years (minimum: 57; maximum: 89). Subjects were divided into two groups: CN (n=66) and AD (n=64).
• **OASIS** (Marcus et al., 2010): This dataset consists of T1w longitudinal scans of 136 subjects from the Open Access Series of Imaging Studies (OASIS-2) project^5^. T1w scans with voxel size of 1×1×1.25 mm were acquired on a 1.5T Siemens Vision scanner using a MP-RAGE sequence. Subjects were scanned on average 2.47 times (minimum: 2 times; maximum: 5 times; 336 scans in total), with a mean interval between scans equal to 702 days (minimum: 182 days; maximum: 1510 days). The mean age at baseline was 75 years (minimum: 60; maximum: 96). Scans were acquired on a Siemens Vision 1.5T scanner. Subjects were divided into two groups: CN (n = 72) and AD (n = 64).
• **OASIS-TR** (Marcus et al., 2010): This dataset consists of the same subjects of the OASIS dataset plus 14 additional subjects from the same project that were diagnosed as converted, for a total of 150 subjects. For each subject, 3 or 4 individual T1w MRI scans obtained in single scan sessions were acquired, for a total of 1845 scans.
• **Munich-TR** (Biberacher et al., 2016): This dataset consists of longitudinal T1w and FLuid Attenuation Inversion Recovery (FLAIR) scans of 2 MS subjects. For each subject, 6 repeated scans were acquired from 3 different 3T scanners (Philips Achieva; Siemens Verio; GE Signa MR750) within 3 weeks (mean interval between successive scans equal to 3 days (minimum: 2 days; maximum: 7 days). Voxel sizes for the T1w scans were 1×1×1 mm for the GE scanner, 1×1×1 mm for the Philips scanner and 1.1×1.1×1 mm for the Siemens scanner. Voxel sizes for the FLAIR scans were 1×1×1 mm for the GE scanner, 1×1×1.5 mm for the Philips scanner, and 1×1×1 mm for the Siemens scanner. T1w scans were acquired using a IR-FSPGR or MP-RAGE sequence. Two scans of “Subject-1” were excluded from the dataset due to scanning protocol violations (Biberacher et al., 2016), and they are not included in the experiments.
• **Munich**: This dataset consists of longitudinal T1w (MP-RAGE sequence) and FLAIR scans of 200 MS subjects acquired on a Philips Achieva 3T scanner. Subjects were scanned on average 6.45 times (minimum: 2 times; maximum: 24 times; 1289 scans in total), with a mean interval between scans equal to 353 days (minimum: 18 days; maximum: 3287 days) and at least 365 days between the first and last visit. Voxel sizes for the T1w scans were either 0.75×0.75×0.75 or, after a scanner change, 1×1×1 mm, while voxel sizes for the FLAIR scans were either 0.75×0.75×0.75 or 0.9×0.9×1.5 mm, respectively. Because some of the T1w scans have image resolutions that differ between time points of the same subject, we resampled all the T1w scans of this dataset to 1 mm isotropic resolution before using them in the experiments. The mean age at baseline was 40 years (minimum: 18; maximum: 74). At each subject’s visit, an Expanded Disability Status Scale (EDSS) score was assessed (mean: 2.46; minimum: 0; maximum: 8.5). Subjects were divided into two groups: stable (n=100) and progressive (n=100) according to the following three strata criteria (Lorscheider et al., 2016): Patients were classified as progressive if (1) the baseline EDSS score is 0 and there is an increase of 1.5 points from the baseline EDSS score to the last time point EDSS score; (2) the baseline EDSS score is between 1 and 5.5 and there is an increase of 1 point from the baseline EDSS sscore to the last time point EDSS score; (3) the baseline EDSS score is above 5.5 and there is an increase of 0.5 points from the baseline EDSS score to the last time point EDSS score. Patients were classified as stable otherwise.
• **ISBI**: This dataset is the publicly available test set of the MS lesion segmentation challenge that was held at the 2015 International Symposium on Biomedical Imaging Carass et al. (2017). It consists of 14 longitudinal MS cases, scanned on average 4.36 times (minimum: 4 times, maximum: 6 times), separated by approximately one year (mean: 391 days, minimum: 299 days, maximum: 503 days). All images were acquired on the same Philips 3T scanner, using a 3D T1w, T2w, PDw and FLAIR sequence. Voxel sizes were 0.82×0.82×1.17 mm for the T1w scans, and 0.82×0.82×2.22 mm for T2w, PDw, and FLAIR. Images were first preprocessed (inhomogeneity correction, skull stripping, dura stripping, and again inhomogeneity correction – see (Carass et al., 2017) for details). Each T1w baseline image was then registered to a 1 mm MNI template and used as target image for registering successive time point images. Each subject’s lesions were delineated by two different raters on the FLAIR scan, and, if necessary, corrected using the other contrasts. Note that the raters were presented with each scan independently, without aiming for longitudinal consistency in their segmentations. In our experiments we used the input combination T1w-FLAIR, which we have found to lead to good lesion segmentation performance in a previous study Cerri et al. (2021).

## Footnotes

1 https://surfer.nmr.mgh.harvard.edu/fswiki/Samseg

2 https://smart-stats-tools.org/lesion-challenge
